# Integrating Constructed Wetlands, Microbial Fuel Cells, and Microalgal Photobioreactors for Sustainable Piggery Wastewater Treatment

**DOI:** 10.3390/biotech15030046

**Published:** 2026-06-25

**Authors:** Diego de Oliveira Corrêa, Alice Ferreira, Belina Ribeiro, Karan Murthy, Anasuya Ganguly, Srikanth Mutnuri, Luisa Gouveia

**Affiliations:** 1 Bioenergy and Biorefineries Unit, LNEG, National Laboratory of Energy and Geology I.P., Estrada do Paço do Lumiar 22, 1649-038 Lisbon, Portugal; diego.biodoc@gmail.com (D.d.O.C.); alice.ferreira@lneg.pt (A.F.); belina.ribeiro@lneg.pt (B.R.); 2 Water Sanitation and Hygiene Laboratory, Department of Biological Sciences, Birla Institute of Technology and Science, KK Birla Goa Campus, NH 17 B, Zuarinagar 403 726, Goa, India; p20240006@goa.bits-pilani.ac.in (K.M.); ganguly@goa.bits-pilani.ac.in (A.G.); srikanth@goa.bits-pilani.ac.in (S.M.); 3 GreenCoLab—Green Ocean Technologies and Products Collaborative Laboratory, Centro de Ciências do Mar do Algarve, Universidade do Algarve, Campus Gambelas, Edifício 7, 8005-139 Faro, Portugal

**Keywords:** bio-electrochemical systems, bioremediation, *Tetradesmus obliquus*, microalgae, swine wastewater

## Abstract

Pig farming generates high-strength piggery wastewater (PWW) with extreme organic and nutrient concentrations. This research evaluated an integrated treatment system combining Vertical Flow Constructed Wetlands (VFCW), Microbial Fuel Cells (MFC), and Microalgae Photobioreactors (PBR) to enhance resource recovery, evaluate bio-electrochemical activity, and produce microalgal biomass. Findings showed that hydraulic saturation in the VFCW–MFC stage enhanced the open-circuit voltage response, reaching a maximum of 539 mV, indicative of bio-electrochemical activity. The optimized VFCW–MFC configuration, featuring pulsed feeding, achieved removals of total suspended solids (TSS, 83%) and chemical oxygen demand (COD, 69%). This integrated pretreatment mitigated ammonia toxicity and turbidity, enabling the subsequent cultivation of *Tetradesmus obliquus* microalga, reaching biomass yields of 1.1–1.3 g L^−1^ while providing crucial tertiary polishing. Overall, the combined VFCW–MFC–PBR system achieved removal efficiencies exceeding 90% for total Kjeldahl nitrogen (TKN) and approximately 80% for COD. This synergistic approach successfully transforms PWW liabilities into valuable assets, including nutrient-rich biomass and bio-electrochemical activity, underscoring the potential of VFCW–MFC–PBR for sustainable wastewater management.

## 1. Introduction

Modern pig farming represents a pillar of global food security, constituting the second-largest source of animal protein consumed worldwide [1,2]. The expansion of intensive farming systems, driven by the demand for low-cost protein and the rapid growth cycle of animals, nonetheless, generates an environmental liability of critical proportions [3]. The annual production of approximately 11.2 billion tons of piggery wastewater (PWW) presents a difficult challenge [4]. This effluent is characterized by an extremely high organic load, toxic concentrations of ammonia, and the presence of emerging contaminants such as antibiotic residues [1]. The improper management of PWW results in severe environmental impacts, including the eutrophication of water bodies, contamination of groundwater, and the emission of potent greenhouse gases like methane and nitrous oxide [1,5]. Furthermore, the dark color and high turbidity of PWW inhibit light penetration, and its high ammonia concentration is toxic to many microorganisms, hindering biological treatment processes [6,7].

In this context, decentralized and low-cost treatment technologies are crucial for the sector’s sustainability. Constructed Wetlands (CWs) are a bio-ecological engineering solution that mimics the purification processes of natural wetlands [8]. By leveraging the synergy among vegetation, substrate, and associated microorganisms, CWs are effective in treating various wastewaters, including domestic, industrial, and leachate effluents [9], efficiently removing organic and inorganic pollutants, nitrogenous compounds, and pharmacological contaminants [10,11]. Among their configurations, Vertical Flow Constructed Wetlands (VFCWs) offer higher efficiency within a smaller footprint by combining physical, chemical, and biological remediation processes [12,13]. Despite their robustness and lower cost, CWs have limitations, such as considerable land area requirements and challenges in the simultaneous and efficient removal of nitrate, total ammoniacal nitrogen, and phosphorus [13].

To overcome these limitations and add value to the process, the integration of bio-electrochemical technologies represents an innovative alternative. Microbial Fuel Cells (MFCs) are devices that utilize the catabolic activity of bacteria to simultaneously oxidize organic matter and generate electricity. In an integrated Constructed Wetland-Microbial Fuel Cell (CW-MFC) system, the natural redox gradient between the lower anoxic zone (anode) and the aerobic rhizosphere (cathode) is harnessed to convert the chemical energy of pollutants into electrical energy [14,15]. This approach not only generates low-power energy, suitable for powering wireless sensors in the field [16], but also accelerates pollutant degradation and mitigates methane emissions [5]. Studies demonstrated the viability of CW-MFCs for PWW, achieving 75.5% Chemical Oxygen Demand (COD) removal with stable bioelectricity production [17]. However, challenges for the widespread application of MFCs include implementation costs and scalability.

Even after advanced treatment with CW-MFC systems, the effluent may still contain nutrients like nitrogen and phosphorus at concentrations requiring a polishing step. For this purpose, microalgae cultivation in photobioreactors (PBRs) presents a sustainable solution. Microalgae assimilate these residual nutrients into their biomass through photosynthesis, a process that additionally sequesters carbon dioxide [18]. Pretreatment with a CW-MFC is crucial, as it reduces the ammonia toxicity and turbidity of the effluent to levels that permit algal growth [19,20]. The primary advantage of this final step is the transformation of a nutrient-laden waste stream into high-value-added biomass, which can be used as a biofertilizer, animal feed, or feedstock for biofuels and other bioproducts [21,22]. The synergistic combination of these three technologies into an integrated hybrid system offers a holistic approach to wastewater management, optimizing resource recovery and energy generation [23]. In this study, a sequential system utilizing CW and MFC, separately or in combination (CW-MFC), was evaluated as a pretreatment step for the subsequent cultivation of microalgae in a PBR. The main objective was to compare the performance of each configuration on piggery wastewater treatment efficiency and the final algal biomass productivity. The proposed optimized system is intended for implementation at a small-scale farm in Goa, India, within the framework of the bilateral Portugal–India project WCAlgaeKIT+ (DRI/India/0609/2020), serving as a model for sustainability and the circular economy in the agricultural sector.

## 2. Materials and Methods

### 2.1. Piggery Wastewater

The piggery wastewater (PWW) was collected from the stabilization lagoon at the Herdade do Pessegueiro property in Glória do Ribatejo, Portugal (39°00′09.000″ N, 8°38′45.500″ W). The PWW corresponded to the liquid fraction of the mixture from pig excreta and water used to clean the housing facilities that accommodate the animals. Table 1 presents the composition of the two PWW batches that were collected to conduct this study and were used for the first and second assays, respectively.

### 2.2. VFCW System Setup

**Figure 1:**
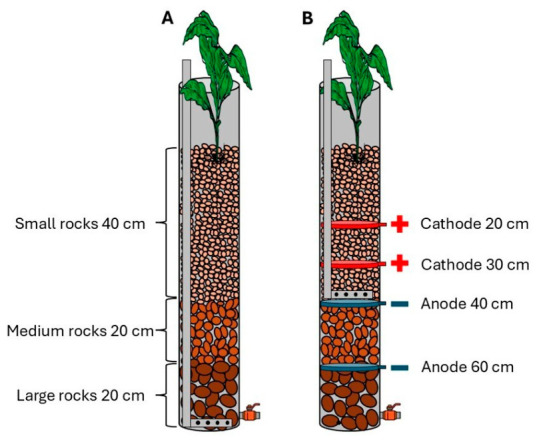
Schematic representation and setup of the Vertical Flow Constructed Wetland (VFCW) coupled with Microbial Fuel Cell (MFC) systems. (**A**) Internal structure of each VFCW unit, showing the stratified substrate layers composed of stones (25–45 mm), medium gravel (10–20 mm), and fine gravel (2–6 mm). Aeration tube at the bottom of VFCW. (**B**) VFCW–MFC unit, showing stainless-steel cathodes (20 and 30 cm depth) and aluminum anodes (40 and 60 cm depth).

The VFCW system was designed by connecting two cylindrical tanks in series [24]. The first (top) tank was placed above the second one so that the liquid could flow from one tank to the other by gravity. The tanks were made of polyvinyl chloride (PVC), with dimensions of 0.20 × 1.0 m (diameter × height), and a sampling valve near the base. The filter materials consisted of stones and gravel of different sizes, which were filled in the following order from bottom to top: 20 cm of large stones (25–45 mm), 20 cm of medium stones/gravel (10–20 mm), and 40 cm of gravel (2–6 mm) (Figure 1A). The VFCW–MFC consists of VFCW with 2 cathodes (stainless-steel at 20 and 30 cm from the top) and 2 anodes (aluminum at 40 and 60 cm from the top) (Figure 1B). All the electrodes had a diameter of 13 cm and were made with a double-welded layer to improve the contact surface with the medium. One *Canna indica* rhizome bearing a 40 cm aerial shoot was planted in each VFCW at a depth of 10 cm. As *C. indica* is a rhizomatous perennial species, a single planted rhizome can produce multiple shoots and an extensive interconnected root system, enabling effective colonization of the available substrate [25]. Passive aeration was provided by a PVC tube (2.5 cm diameter) positioned at the base of the normal VFCW and at a depth of 40 cm in the VFCW–MFC. Two different trials were made to evaluate the operating parameters.

### 2.3. Experimental Design and Optimization Strategy of the VFCW–MFC System

The experimental work was designed as a sequential optimization process to investigate the influence of hydraulic and operational conditions on the performance of integrated VFCW–MFC systems treating piggery wastewater. Rather than isolating the contribution of individual factors, the study progressively evaluated how different design and operational strategies affected pollutant removal and the electrochemical response of the integrated system. Each hydraulic configuration was represented by a single VFCW–MFC unit, allowing the evaluation of a broad range of operational conditions and their influence on the integrated treatment process.

The VFCWs were operated under an intermittent feeding regime, with 2 L of piggery wastewater applied daily to the first-stage units at a flow rate of 1 L min^−1^. Feeding was performed at the first-stage units and left to drain throughout the substrate layers. The PWW was allowed to percolate through the substrate layers, and once it reached the bottom of the first-stage units, it was transferred to the corresponding second-stage units by opening the outlet valve, maintaining a flow rate of 1 L min^−1^. After passing through the second stage, the effluent volume was collected through the base valves for further analysis. Under unsaturated operation, the wastewater was allowed to drain freely through the wetland media, such that the effective contact time corresponded to the drainage period through the substrate. In contrast, under saturated operation, the wastewater was retained within the pore volume of the second-stage units between feeding events and discharged immediately before the next daily feeding cycle, corresponding to an approximate contact time of 24 h. The same feeding schedule and operational protocol were maintained throughout the experiments to ensure consistent hydraulic conditions and comparable reactor performance among configurations.

#### 2.3.1. First Assay: Effect of Aeration and Hydraulic Saturation

**Figure 2:**
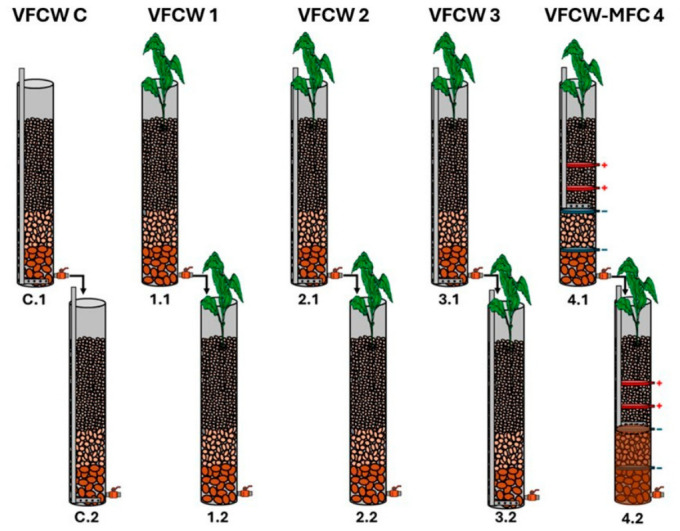
Schematic representation of the experimental configurations of Vertical Flow Constructed Wetland (VFCW) systems during the first trial. Each system consists of two stages—the first stage (**top column**) and the second stage (**bottom column**): VFCW C (C.1–C.2)—both stages operated without plants and with passive aeration (Control); VFCW 1 (1.1–1.2)—both stages planted and operated without aeration; VFCW 2 (2.1–2.2)—first stage (2.1) planted with passive aeration, followed by a planted second stage without aeration (2.2); VFCW 3 (3.1–3.2)—both stages planted and operated with passive aeration; VFCW–MFC 4 (4.1–4.2)—both stages planted, operated with passive aeration, and equipped with a microbial fuel cell (MFC). Only the second stage of VFCW–MFC 4 (4.2) was saturated with piggery wastewater.

The first trial evaluated the role of passive aeration versus anaerobic conditions and the effect of PWW saturation on treatment performance and the bio-electrochemical activity of the VFCW–MFC systems. The experimental setup consisted of: C.1/C.2 (control), passive aeration and no planting; 1.1/1.2, no aerated and planted; 2.1, aerated and 2.2, no aerated, both planted; 3.1/3.2, both aerated and planted; 4.1/4.2, both VFCW–MFC planted and aerated—4.2 was the only one saturated with PWW at 10 cm from the top (Figure 2). All systems were fed daily with 2 L of PWW. After passing through the lower VFCWs, the total effluent volume was collected through the base valves for further analysis and microalgal cultivation, except VFCW–MFC 4.2, which was maintained saturated with PWW. The bio-electrochemical response of the VFCW–MFC systems was evaluated by monitoring the open-circuit voltage (OCV), which was used as an indicator of bio-electrochemical activity and redox stratification rather than as a direct measure of recoverable electrical power. OCV measurements were performed for all cathode-anode combinations (20–40 cm, 20–60 cm, 30–40 cm, and 30–60 cm, respectively).

#### 2.3.2. Second Assay: Effect of Hydraulic Configuration and Feeding Regime

**Figure 3:**
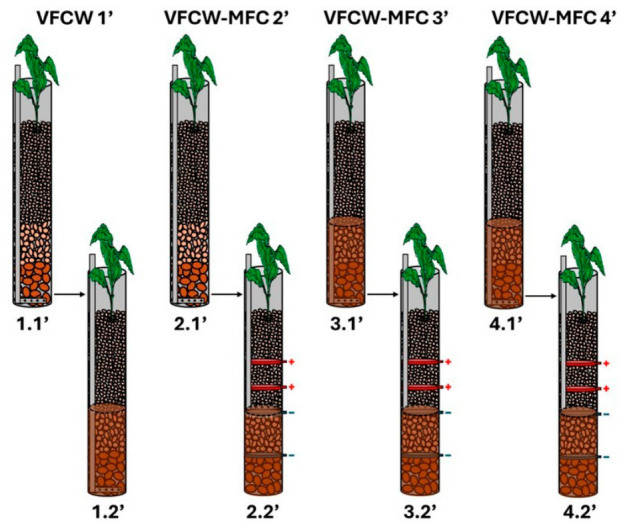
Schematic representation of the experimental configurations of Vertical Flow Constructed Wetland (VFCW) coupled with Microbial Fuel Cell (MFC) systems during the second trial. Each system consists of two stages—the first stage (**top column**) and the second stage (**bottom column**): VFCW 1′ (1.1′–1.2′)—first stage operated under unsaturated conditions (1.1′), followed by a saturated second stage without MFC (1.2′); VFCW–MFC 2′ (2.1′–2.2′)—first stage unsaturated (2.1′), followed by a saturated second stage equipped with an MFC (2.2′); VFCW–MFC 3′ (3.1′–3.2′)—both stages operated under saturated conditions, with an MFC installed in the second stage (3.2′), fed with a single pulse of 2 L. VFCW–MFC 4′ (4.1′–4.2′)—same configuration as VFCW–MFC 3′, but fed in a fractionated mode (4 × 500 mL). All systems were planted and operated under passive aeration.

Based on the findings of the first assay, which identified hydraulic saturation as a key factor for establishing favorable redox conditions, a second assay was designed to further optimize the integrated VFCW–MFC system. The objective was to evaluate how progressively modified hydraulic configurations and feeding strategies influenced the treatment performance and bio-electrochemical response of the system. Four configurations were tested: VFCW 1′, comprising an unsaturated first stage (1.1′) followed by a saturated second stage without MFC (1.2′); VFCW–MFC 2′, with an unsaturated first stage (2.1′) followed by a saturated MFC stage (2.2′); VFCW–MFC 3′, with both stages operated under saturated conditions and the MFC installed in the second stage, receiving a single daily 2 L pulse; and VFCW–MFC 4′, identical to VFCW–MFC 3′ but operated under a fractionated feeding regime (4 × 500 mL). All systems were planted and operated under passive aeration (Figure 3).

The second assay progressively evaluated the influence of (i) introducing a saturated second stage, (ii) extending saturation to both treatment stages, and (iii) implementing a fractionated feeding strategy while maintaining the same daily hydraulic loading.

### 2.4. Microalgal Growth and Secondary Wastewater Treatment

Following treatment through the integrated system, passing sequentially through the upper and lower VFCWs, the effluent was collected to serve as the culture medium for the microalga *Tetradesmus obliquus* (ACOI 204/07, ACOI Culture Collection, University of Coimbra, Coimbra, Portugal). Microalgal cultivations were conducted using 100% effluent, supplemented only by the inoculum volume (10% *v*/*v*). The 1 L Schott flasks, acting as bubble-column photobioreactors (PBRs), were maintained in a climate-controlled room at a temperature of 25 ± 1 °C, under constant illumination of 52 µmol m^−2^ s^−1^ and an aeration rate of 1 vvm, providing mixing and carbon supply for microalgal growth. Biomass production was monitored for four weeks by determining the ash-free dry weight of cultures. Experiments were performed using two biological replicates.

### 2.5. Sampling and Analytical Methods

To monitor the system efficiency, post-treatment effluent samples were collected weekly from all VFCWs. Pollutant analysis was performed according to standard methods: Total suspended solids (TSS, 2540D) and volatile suspended solids (VSS, 2540D), chemical oxygen demand (COD, 5220B), total Kjeldahl nitrogen (TKN, 4500-Norg B), ammonia nitrogen (TAN, 4500-NH_3_) [26]. Nitrate concentration was determined based on a calibration curve prepared using sodium nitrate within the range of 25–500 mg NO_3_^−^ L^−1^. The absorbance was measured in a dual-beam UV-Vis spectrophotometer (Hitachi U-200, Tokyo, Japan) and calculated as the difference between readings at 220 nm and twice the absorbance at 275 nm. Phosphate was quantified using the Hach PhosVer^®^ 3 ascorbic acid method (powder pillow), with a measuring range of 0.02–2.50 mg PO_4_^3−^ L^−1^, by reading the absorbance in a direct reading spectrophotometer (Hach DR/2000, Loveland, CO, USA). pH was determined using a pH meter (WTW inoLab Level 1, Weilheim, Germany). The OCV was measured using a digital multimeter (Lexman model LX-M 2000, ADEO, Lezennes, France) connected to the various cathode-anode combinations (20–40 cm, 20–60 cm, 30–40 cm, and 30–60 cm, respectively), in intervals of 2 h starting immediately after feeding until reaching 8 h (0, 2, 4, 6, and 8 h). All measurements were performed in duplicate.

### 2.6. Statistical Analysis

For the constructed wetland assays, statistical comparisons were based on weekly measurements collected during stable reactor operation. Data are reported as median, interquartile range (IQR: 25th–75th percentile), minimum, and maximum values to provide a robust descriptive characterization of central tendency and variability across reactor configurations. No inferential comparisons were made for these datasets, and the observed differences among reactors are discussed as exploratory trends.

For the microalgae assay, normality and homogeneity of variances were assessed using the Shapiro–Wilk test. When assumptions were met, differences among treatments were evaluated by one-way ANOVA followed by Dunnett’s post hoc tests, adopting *p* ≤ 0.05 as the significance threshold. All statistical analyses were performed using GraphPad Prism 8.0.1.

## 3. Results and Discussion

### 3.1. Effect of Saturation on Treatment Efficiency and Bio-Electrochemical Response

**Figure 4:**
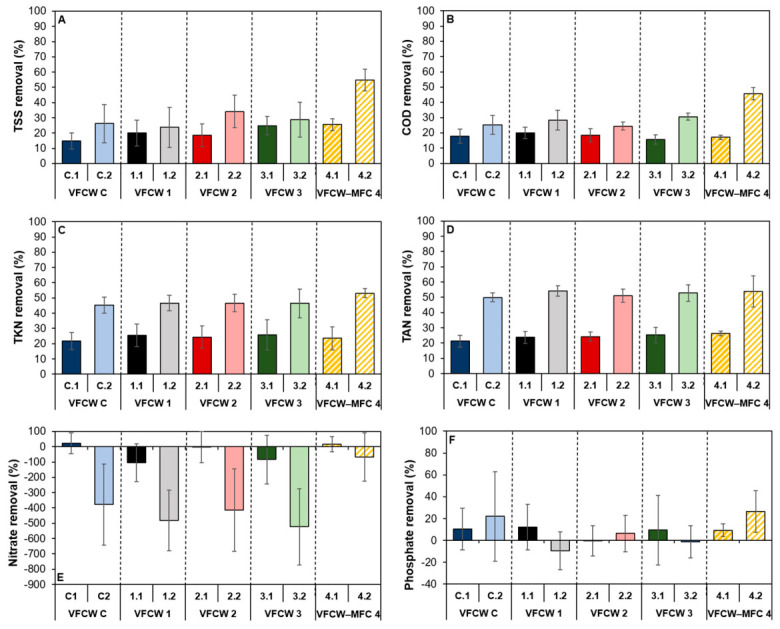
Pollutant removal efficiencies of piggery wastewater treated by Vertical Flow Constructed Wetland (VFCW) systems in the first assay. (**A**) Total suspended solids (TSS); (**B**) chemical oxygen demand (COD); (**C**) total Kjeldahl nitrogen (TKN); (**D**) total ammonia nitrogen (TAN); (**E**) nitrate; (**F**) phosphate. VFCW configurations: VFCW C (C.1–C.2)—unplanted with passive aeration (Control); VFCW 1 (1.1–1.2)—both stages planted and non-aerated; VFCW 2 (2.1–2.2)—planted and aerated first stage (2.1) followed by a planted, non-aerated second stage (2.2); VFCW 3 (3.1–3.2)—both stages planted and aerated; VFCW–MFC 4 (4.1–4.2)—both stages planted, aerated, and integrated with microbial fuel cells (MFCs). Only unit 4.2 was operated under saturated conditions. Values are given as the average over the 4-week assay.

**Figure 5:**
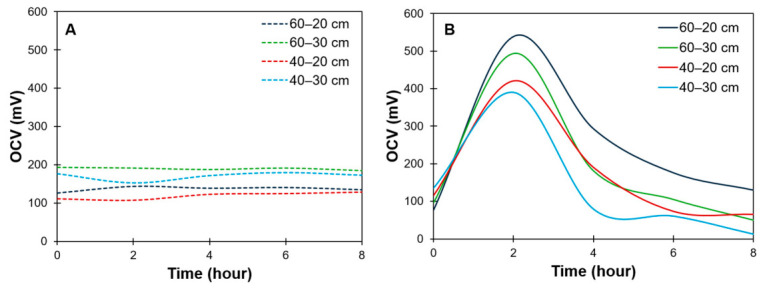
Open-circuit voltage (OCV) profiles from the first assay for the Vertical Flow Constructed Wetland-Microbial Fuel Cell (VFCW–MFC) 4: (**A**) 4.1 (unsaturated condition); (**B**) 4.2 (saturated condition). Electrode spatial zonation: anode (40 and 60 cm from the top); cathode (20 and 30 cm from the top).

The primary objective of the first assay was to evaluate how hydraulic saturation affects both contaminant removal and bio-electrochemical performance in a VFCW–MFC configuration. The results suggest that saturation substantially influenced substrate redox stratification, with apparent effects on both physical-biological removal mechanisms (Figure 4) and open-circuit voltage (OCV) generation (Figure 5).

TSS and COD removals were substantially more effective under saturated conditions (VFCW–MFC 4.2), with median reductions of 51.9% (IQR: 47.4–56.2%) and 47.0% (IQR: 40.5–49.9%), respectively, compared to 26.9% (IQR: 17.4–32.8%) and 17.0% (IQR: 15.5–18.8%) under unsaturated conditions (VFCW–MFC 4.1) (Figure 4A,B). Second-stage reactors 2.2 and 3.2 showed intermediate TSS removal, with medians of 31.7% (IQR: 26.2–42.5%) and 25.6% (IQR: 23.8–32.8%), respectively, while for COD, reactors 3.2 and 1.2 stood out among second-stage units, recording medians of 30.8% (IQR: 27.8–33.4%) and 26.2% (IQR: 21.1–36.3%), indicating a cumulative effect of the sequential treatment combined with passive aeration on organic matter degradation. These results demonstrate that, although mechanical filtration in vertical downward flow provides basal TSS removal, the combined effects of permanent saturation, the electrochemical activity of the MFC, and the development of stable biofilms within the VFCW–MFC systems were likely the main factors contributing to the enhanced removal of both parameters. In VFCW systems, TSS reduction occurs primarily through physical processes of mechanical filtration and sedimentation within the substrate matrix, with efficiency directly influenced by filter material granulometry. Finer particles enhance retention capacity, though they increase long-term clogging risk [15,26,27,28]. Beyond physical barriers, adhesion of particles to microbial biofilms coating the substrate and stabilization provided by plant roots are essential complementary mechanisms for effective solids capture [15,27,28]. *Canna indica*, the plant used in this study, is highly relevant for particle filtration in CWs because its dense fibrous root system enhances physical filtration and sediment trapping, while also increasing the surface area for biofilm development and suspended solids retention within the rhizosphere [25,29,30].

The saturated stage (VFCW–MFC 4.2) provided extended and continuous contact between PWW and biofilms, favored by prolonged wastewater-substrate contact under saturated conditions, and increased effective hydraulic residence within the media, stabilizing the anoxic zones within the substrate and promoting anaerobic degradation pathways (fermentation and subsequent consumption of intermediates by anaerobic consortia) that would be suppressed in predominantly aerobic systems [31,32]. Physical retention of fine solids and colloids was further enhanced by saturation, contributing to greater TSS and particulate COD removal. These observations align with established understanding in CW and hybrid CW–MFC systems, where treatment efficiency emerges from combined filtration, sorption, and biodegradation, all strongly modulated by hydraulic regime, redox distribution, and biogeochemical environment defined by saturation and retention time [33,34,35].

Nitrogen transformation was highly dependent on process staging. TKN removals were markedly higher in second-stage reactors, with medians ranging from 43.9% (IQR: 40.7–51.6%) in C.2 to 54.1% (IQR: 51.0–55.5%) in 4.2, compared to first-stage units, which ranged from 19.9% (IQR: 16.7–27.2%) in C.1 to 26.4% (IQR: 14.4–34.3%) in 3.1. Similarly, TAN removals followed the same two-stage pattern, with second-stage medians ranging from 50.2% (IQR: 47.1–53.1%) in C.2 to 55.6% (IQR: 41.5–63.8%) in 4.2, versus 20.7% (IQR: 17.4–25.8%) to 26.4% (IQR: 25.0–28.1%) in first-stage units. These results demonstrate that sequential two-stage treatment was the overriding factor governing nitrogen removal, with no consistent differentiation among configurations within each stage. Effluent nitrate data confirmed net NO_3_^−^ accumulation through nitrification in most second-stage reactors (medians: −270 to −524 mg L^−1^ relative change), consistent with predominant nitrification under aerobic conditions. Notably, reactor 4.2 exhibited a median near zero (−31 mg L^−1^, IQR: −212–62 mg L^−1^), indicating a closer balance between nitrification and nitrate consumption. This pattern is consistent with enhanced nitrate consumption in anoxic microsites established under saturated conditions, possibly influenced by the presence of the MFC, although direct evidence from intermediate compound measurements (NO_2_^−^, N_2_, N_2_O) is not available to confirm these pathways. Under intermittent unsaturated feeding, passive aeration may favor nitrification while reducing prolonged microbial–substrate contact; in contrast, the saturated stage (VFCW–MFC 4.2) presumably provided more stable anoxic microsites, enabling nitrate removal via denitrification when residual carbon remained available [31,32]. These observations suggest a functional separation between treatment stages—the first stage likely promoting ammonia oxidation under predominantly aerobic conditions, and the second saturated stage potentially facilitating subsequent nitrate removal—consistent with mechanisms reported for analogous CW–MFC systems [31,32]. However, the absence of nitrification/denitrification intermediate measurements means that these mechanistic interpretations should be regarded as hypotheses requiring further experimental validation.

Phosphorus removal was highly variable across all units (Figure 4F), with net release observed across most configurations, as evidenced by negative or near-zero median removals in most reactors (range: −5.1% to 19.3%). The wide interquartile ranges observed across all units, notably in C.2 (IQR: −6.0–61.9%) and 1.2 (IQR: −28.3–4.1%), reflect high temporal variability and the absence of a consistent removal trend attributable to any specific hydraulic or operational configuration. The highest median removal was recorded in reactor 4.2 (19.3%; IQR: 10.7–45.8%), the only saturated unit, though the overlapping IQRs across reactors preclude any mechanistic attribution. These results suggest that PO_4_^3−^ dynamics were governed primarily by physicochemical processes intrinsic to the CW substrate rather than by reactor configuration. According to previous studies, P retention in VFCWs is predominantly regulated by finite and reversible substrate adsorption and precipitation processes [36,37], consistent with the progressive depletion of P retention capacity observed here. Previous studies have shown that phosphorus accumulation in gravel-based CWs is finite, with increased phosphorus mobility reported after prolonged loading and accumulation levels on the order of 100–300 mg P kg^−1^ [38,39].

Additionally, the potential release of Al^3+^ ions through anodic dissolution of the aluminum electrodes used in the VFCW–MFC configuration cannot be excluded under the reducing conditions of the saturated substrate zone. Given that Al^3+^ may react with soluble phosphate to form AlPO_4_ under the near-neutral to alkaline pH conditions observed (pH 7.99–8.15), an abiotic precipitation pathway may have contributed to the phosphate removals recorded in this configuration. In the absence of dissolved aluminum measurements, this represents an unresolved confounding factor in the interpretation of phosphorus dynamics.

The pH values remained stable and slightly alkaline throughout the experiment (7.99–8.15), indicating adequate buffering capacity and absence of limiting acidic or alkaline conditions. The marginally lower pH in the saturated unit (VFCW–MFC 4.2, pH = 7.99) compared to the unsaturated counterpart (VFCW–MFC 4.1, pH = 8.15) likely results from combined (i) enhanced organic matter oxidation and associated CO_2_ production in the anodic compartment and (ii) the opposing effects of nitrification and denitrification, since nitrification is acidifying, while denitrification contributes to alkalinity recovery. Nevertheless, the observed pH range remained favorable for microbial activity and the stable operation of both wetland and bio-electrochemical processes, as commonly reported for CW–MFC systems [40].

Corrêa et al. [24] reported removal efficiencies for COD (20.5%), TSS (11.6%), TKN (42.9%), and TAN (50.3%) that are significantly lower than those achieved in the present study, which reached values of 45.5%, 54.7%, 53.1%, and 53.8%, respectively. These improved results are attributed to the implementation of media saturation and the transition to passive aeration, which effectively addressed the operational limitations identified in the previous study. While unsaturated gravity flow in that previous study restricted the contact time between the effluent and the biofilm, the saturation employed in the current work facilitated continuous and prolonged contact, stabilizing anoxic zones and enhancing the physical filtration capacity for fine solids. Furthermore, the elimination of active mechanical aeration, which previously induced detrimental turbulence and the subsequent resuspension of solids and biofilms, in favor of a passive aeration system preserved the integrity of the microbiota and ensured the maintenance of vertical redox gradients essential for the superior efficiency of organic degradation and nitrogen transformation. This option also decreases the energy expenses for aeration. In addition, *Canna indica*, the plant used in the present study, generally outperforms ornamental species such as lucky bamboo (*Dracaena sanderiana*) due to its denser fibrous root system, higher rhizosphere surface area, and stronger oxygen release, which enhance microbial activity, filtration, and nutrient removal [25].

After quantifying pollutant removal patterns, it became evident that hydraulic saturation was decisive for establishing robust redox gradients and, consequently, for enhancing the bio-electrochemical response of the VFCW–MFC systems, as reflected by elevated OCV values [41]. The unsaturated stage (Figure 5A) exhibited low and relatively constant OCV (~107–194 mV), consistent with greater oxygen availability acting as a competing electron acceptor and limiting the electron fraction redirected toward the anode. By contrast, the saturated stage (Figure 5B) displayed a classical post-feeding response, with OCV peaks occurring 2 h after feeding (up to 539 mV for the 60–20 cm electrode arrangement), followed by progressive decline over 8 h. This transient behavior is widely reported in bio-electrochemical systems and reflects rapid oxidation of readily biodegradable organic matter, followed by substrate depletion and reduced electron donor availability [42,43].

Electrode positioning had a marked effect under saturated conditions: the most effective configuration placed the anode at a greater depth (60 cm) and the cathode at a shallower depth (20 cm), maximizing the vertical redox gradient. This arrangement ensured a persistent anoxic zone for the anode whilst providing the cathode with improved oxygen accessibility. This spatial separation between electron donors and acceptors enhanced the electrochemical response by creating a steep redox gradient, with saturation in the second reactor playing a fundamental role in establishing stable redox gradients by minimizing dissolved oxygen intrusion into the anodic region and favoring electron transfer toward the electrode [15,28,32]. Under unsaturated conditions, oxygen competes as a terminal electron acceptor, reducing the OVC response [28]. The OCV values observed here (peaks of 539 mV) fall within the range reported for comparable systems, though absolute comparisons must account for differences in organic loading, electrode materials, operational scale, and biofilm maturity. The temporal OCV dynamics, characterized by a peak shortly after feeding followed by decay, indicate that the electrochemical response was closely linked to substrate availability and microbial metabolic activity [32,44,45].

These findings indicate that saturation in the second stage enhances both treatment performance (especially for COD and TSS, with potential nitrogen cycling improvements due to stable anoxic microsites) and the bio-electrochemical activity of the VFCW–MFC systems by sustaining favorable redox stratification. The OCV decline throughout the cycle suggests that the window of elevated electroactivity is brief and substrate dependent. Literature indicates that operational strategies such as pulsed or intermittent feeding can enhance and extend transient OCV responses by periodically replenishing readily biodegradable substrates, thereby sustaining electron donor availability and promoting repeated electrochemical activation cycles in CW–MFC systems [42,43]. In parallel, cathode positioning plays a critical role in regulating oxygen diffusion and redox stratification, with optimal configurations enhancing voltage stability by maintaining a well-defined aerobic–anaerobic interface [46,47]. Moreover, hydraulic saturation has been shown to improve system performance by stabilizing anaerobic conditions in the anodic region, increasing effective contact time, and strengthening electron transfer pathways, while simultaneously improving pollutant removal [48,49]. Although OCV does not directly quantify recoverable electrical power, the results demonstrate that hydraulic configuration plays a key role in promoting favorable bio-electrochemical conditions within integrated VFCW–MFC systems.

### 3.2. Effects of Saturation and Feeding Regime on Treatment Efficiency and Bio-Electrochemical Response

The second assay evaluated how different hydraulic configurations, including saturation and feeding regime, influenced treatment performance and the OCV response of the integrated VFCW–MFC systems. Only units 1.1′ and 2.1′ operated unsaturated, while all others operated under saturated conditions (Figure 3). Units 4.1′ and 4.2′ received 2 L d^−1^ of PWW fractionated into four daily pulses of 500 mL, whilst others maintained single daily feeding with identical total volume.

**Figure 6:**
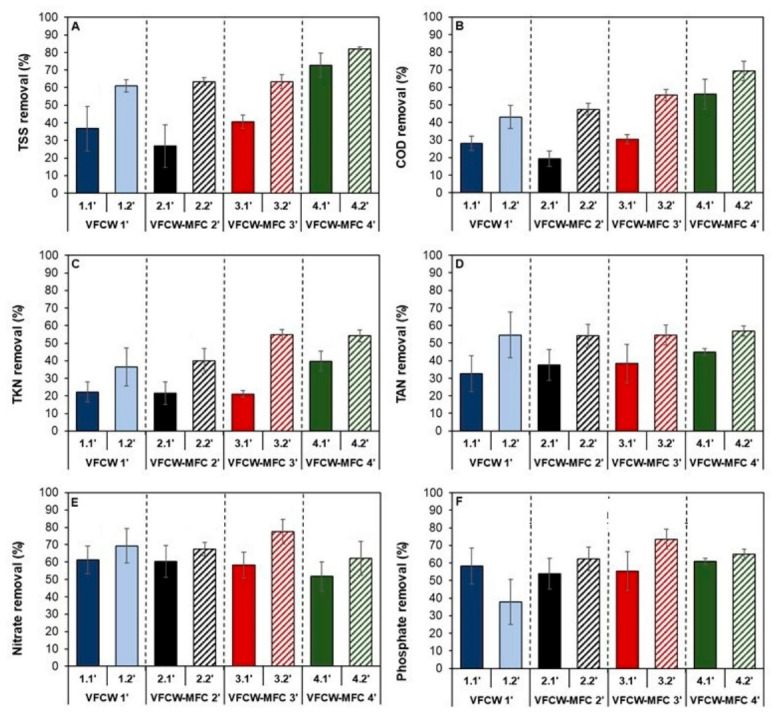
Pollutant removal efficiencies of piggery wastewater treated by Vertical Flow Constructed Wetland (VFCW) coupled with Microbial Fuel Cell (MFC) systems during the second trial. (**A**) Total suspended solids; (**B**) chemical oxygen demand; (**C**) total Kjeldahl nitrogen; (**D**) total ammonia nitrogen; (**E**) nitrate; (**F**) phosphate. VFCW configurations: VFCW 1′ (1.1′–1.2′)—first stage operated under unsaturated conditions (1.1′), followed by a saturated second stage without MFC (1.2′); VFCW–MFC 2′ (2.1′–2.2′)—first stage unsaturated (2.1′), followed by a saturated second stage equipped with an MFC (2.2′); VFCW–MFC 3′ (3.1′–3.2′)—both stages operated under saturated conditions, with an MFC installed in the second stage (3.2′), fed with a single pulse of 2 L. VFCW–MFC 4′ (4.1′–4.2′)—same configuration as VFCW 3′, but fed in a fractionated mode (4 × 500 mL). All systems were planted and operated under passive aeration. Values are given as the average over the 4-week assay.

A systematic performance gradient was observed for TSS removal, increasing from the unsaturated to saturated configurations, with maximum efficiencies achieved under saturated conditions (Figure 6A). Hydraulic saturation likely enhanced solids retention through combined physical filtration and particle capture within water-filled pore spaces, while simultaneously promoting greater biofilm continuity and matrix stability, reducing remobilization of retained particles. In VFCWs, suspended solids are preferentially retained within the upper filter layer, where the interaction between accumulated particles and biofilm development governs both filtration efficiency and hydraulic behavior [50,51].

The pulsed feeding regime applied in VFCW–MFC 4′ likely mitigated hydraulic surges and preferential flow, favoring gradual solids deposition and more stable flow distribution. Both feeding strategy and saturation are known to strongly influence residence time, oxygen transport, and pore-scale hydrodynamics in VFCW systems, thereby modulating both solids capture and biofilm persistence [52,53]. Similar behavior has also been reported in VFCW–MFC configurations, where stable hydraulic conditions promote cohesive biofilm development and improve overall filtration and treatment performance [54,55].

COD and TSS removals followed closely aligned trends, with consistently higher efficiencies in the second stage, underscoring the substantial contribution of particulate and colloidal fractions to overall pollutant removal (Figure 6A,B). Reactor 4.2′ achieved the highest removal for both parameters, with median TSS removal of 81.9% (IQR: 81.4–83.1%) and median COD removal of 69.4% (IQR: 63.1–74.3%), reflecting both superior efficiency and high operational stability. Both 4.2′ and 4.1′ exhibited comparatively high COD and TSS removals, median TSS of 73.2% (IQR: 68.5–78.6%) and median COD of 55.8% (IQR: 47.9–65.2%) for 4.1′, suggesting beneficial interactions among hydraulic saturation, passive aeration, and MFC integration in promoting organic matter degradation and solids retention. The notably wider IQR for COD in 4.1′ compared to 4.2′ indicates that fractionated feeding contributed to greater process stability beyond efficiency gains alone. Second-stage reactors 1.2′, 2.2′, and 3.2′ showed similar median TSS removals of 61.3% (IQR: 59.7–63.3%), 62.9% (IQR: 62.5–64.4%), and 65.0% (IQR: 61.6–65.3%), respectively, and COD removals of 42.6% (IQR: 38.5–49.2%), 47.3% (IQR: 45.3–49.6%), and 55.5% (IQR: 52.1–59.0%), respectively, with consistently narrow IQR values, reinforcing the benefit of sequential treatment regardless of first-stage configuration. Conversely, reactors lacking MFC and aeration (1.1′ and 2.1′) exhibited the lowest median removals for both parameters—TSS: 40.2% (IQR: 31.0–44.3%) and 22.0% (IQR: 21.2–32.1%); COD: 27.9% (IQR: 24.5–31.9%) and 18.4% (IQR: 15.7–23.5%), respectively, alongside higher variability, indicating that these features are critical drivers of treatment efficiency in the CW system. Beyond physical retention, saturation extended contact time and stabilized anoxic zones, promoting fermentative and anaerobic pathways that complemented oxygenated microsites. In VFCW–MFC configurations, this redox stratification sustains electroactive communities surrounding the anodes, yielding superior performance in saturated setups such as configuration 4.2, which was optimized through fractionated feeding regimes [32,33].

TKN removals varied with bed maturation and hydraulic regime, with peak efficiencies observed in the fully saturated second-stage reactors (Figure 6C). Reactors 3.2′ and 4.2′ achieved the highest median TKN removals, 54.4% (IQR: 52.2–57.4%) and 54.0% (IQR: 51.9–56.7%), respectively, with comparably narrow IQR values, indicating consistent performance under both single-pulse and fractionated feeding strategies. Reactors 2.2′ and 4.1′ occupied an upper-intermediate position, with median removals of 38.0% (IQR: 34.0–48.1%) and 41.7% (IQR: 33.7–44.2%), respectively, suggesting that both second-stage saturated treatment and first-stage MFC configuration contribute meaningfully to nitrogen removal. Reactor 1.2′ exhibited transitional behavior, with a median of 34.2% (IQR: 25.3–50.0%) and the widest IQR among second-stage units, reflecting greater performance variability likely associated with the absence of MFC integration. First-stage reactors 1.1′, 2.1′, and 3.1′ recorded the lowest TKN removals, medians of 19.2% (IQR: 18.0–25.7%), 19.7% (IQR: 16.0–28.2%), and 21.4% (IQR: 18.9–22.5%), respectively. This confirms that the absence of passive aeration, MFC, and sequential treatment limits TKN removal capacity. The high TKN removals observed in configurations 3.2′ and 4.2′ suggest that integrated hydraulic operation, including saturation and two-stage treatment, promoted favorable conditions for coupled nitrification–denitrification [32,56]. However, these transformation pathways remain hypothetical in the absence of measurements of nitrogen intermediates (NO_2_^−^, N_2_, N_2_O), as previously stated. Fractionated feeding in configuration 4′ may have contributed to maintaining carbon availability and hydraulic stability, although its individual contribution cannot be isolated from the other operational factors.

TAN removals ranged from 19.2 to 54.4% (median) across reactor configurations in Assay 2 (Figure 6D). First-stage reactors (1.1′ and 2.1′) showed the lowest and most variable removals, with medians of 19.2% (IQR: 18.0–25.7%) and 19.7% (IQR: 16.0–28.2%), respectively, suggesting that a single passage without complementary treatment components was insufficient to enhance TAN removal. Second-stage reactors generally achieved higher removals: 3.2′ reached a median of 54.4% (IQR: 52.2–57.4%), and 4.2′ reached 54.0% (IQR: 51.9–56.7%), while 1.2′ and 2.2′ attained intermediate values of 34.2% (IQR: 25.3–50.0%) and 38.0% (IQR: 34.0–48.1%), respectively. This broad performance gradient suggests that the combined hydraulic and operational conditions, including saturation regime, passive aeration, plant activity, and MFC integration, likely contributed to maintaining localized oxic and anoxic microsites [34,36].

Nitrate removal was consistently achieved across all configurations (Figure 6E). Reactor 3.2′ attained the highest median removal (72.5%; IQR: 63.6–83.8%), followed by 4.2′ (67.1%; IQR: 54.3–74.8%) and 2.1′ (59.5%; IQR: 39.9–65.2%), while 1.2′ showed the lowest and most variable median removal (26.5%; IQR: 23.7–57.6%), indicating inconsistent denitrification performance in this unit. Reactors 4.1′ and 4.2′ presented similar median removals (59.5% and 67.1%, respectively), with overlapping IQRs, reinforcing the hypothesis that simultaneous cathodic denitrification may have limited net NO_3_^−^ removal in these MFC-integrated units. The slightly lower and more variable performance observed in VFCW–MFC 4′ likely reflects reduced instantaneous carbon availability for denitrifiers under fractionated feeding, despite improved hydraulic stability [32,36].

Phosphate removal showed substantial variability across reactor configurations, with medians ranging from 26.5% (1.2′; IQR: 23.7–57.6%) to 72.5% (3.2′; IQR: 63.6–83.8%) (Figure 6F). Despite these numerical differences, the wide and largely overlapping IQRs across most reactors suggest that PO_4_^3−^ removal was primarily governed by abiotic mechanisms largely independent of reactor configuration, consistent with the finite adsorption and precipitation dynamics characteristic of VFCW substrates [40]. In the conventional VFCW 1′, a marked contrast was observed between stages: the unsaturated first stage (1.1′) reached a median removal of 50.0% (IQR: 41.9–78.6%), while the saturated second stage (1.2′) declined to 26.5% (IQR: 23.7–57.6%), with a notably wide range (21.1–75.1%) reflecting high temporal variability. This pattern likely reflects the gradual depletion of phosphorus-binding capacity in the substrate, since phosphate retention in VFCWs primarily occurs through finite adsorption and precipitation mechanisms [40].

Higher and more stable phosphate removals were observed in the saturated VFCW–MFC configurations: 2.2′ reached a median of 59.0% (IQR: 57.4–69.0%), 3.2′ attained 72.5% (IQR: 63.6–83.8%), and 4.2′ maintained 67.1% (IQR: 54.3–74.8%) under fractionated feeding. Notably, 3.2′ also presented the narrowest range among high-performing units, suggesting more consistent performance. Recent studies report that CW–MFC systems can improve phosphorus retention through intensified redox stratification, Fe cycling, and additional reactive surfaces provided by electrodes and electrogenic biofilms [40,57]. However, given the broad IQR overlaps among reactors, these trends cannot be unequivocally attributed to MFC integration, hydraulic saturation, or feeding strategy, but rather to the physicochemical properties of the substrate, as previously stated. Although numerical differences among treatments were observed, they should be regarded as exploratory trends that warrant further investigation using replicated reactor systems. Additionally, as previously discussed, the potential contribution of Al^3+^-driven phosphate precipitation through anodic dissolution of the aluminum electrodes also cannot be excluded in the VFCW–MFC configurations of this assay and represents an additional source of uncertainty in the attribution of phosphorus removal trends.

The results for piggery wastewater treatment in the second assay are significantly superior to those reported by Corrêa et al. [24], demonstrating the benefits of the combined hydraulic and operational modifications implemented in the present study, including media saturation and fractionated feeding. The daily feeding regime consisting of four pulses attenuated hydraulic peaks and facilitated incremental solids deposition. Passive rather than active aeration eliminated particle resuspension observed in the previous study, leading to inconsistent pollutant removal, especially for phosphate.

These findings underscore the importance of hydraulic configuration in establishing complementary aerobic and anoxic niches that optimize organic matter and nutrient removal through integrated biological and physicochemical pathways [32,35,56].

**Figure 7:**
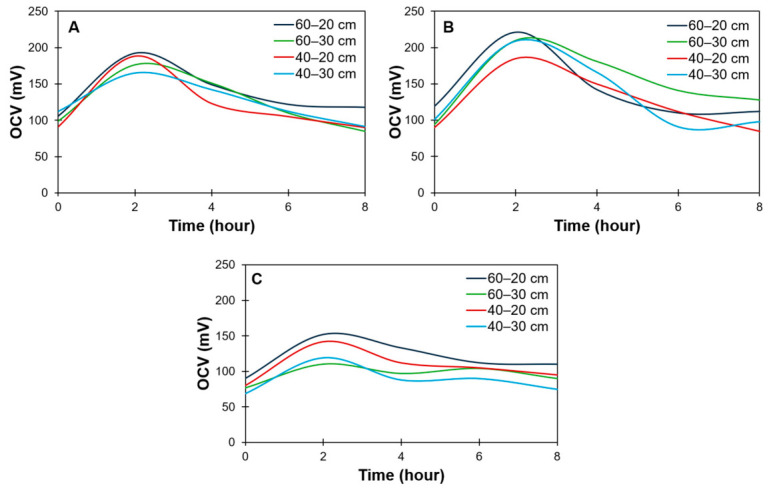
Open-circuit voltage (OCV) profiles in the second assay for Vertical Flow Constructed Wetland-Microbial Fuel Cell (VFCW–MFC) units: (**A**) VFCW–MFC 2′ (2.1′–2.2′)—first stage unsaturated (2.1′), followed by a saturated second stage equipped with an MFC (2.2′); (**B**) VFCW–MFC 3′ (3.1′–3.2′)—both stages operated under saturated conditions, with an MFC installed in the second stage (3.2′), fed with a single pulse of 2 L, and (**C**) VFCW–MFC 4′ (4.1′–4.2′)—same configuration as VFCW 3, but fed in a fractionated mode (4 × 500 mL). Electrode spatial zonation: anode (40 and 60 cm from top); cathode (20 and 30 cm from top).

Building upon the observation from the first assay, this second experiment confirmed that hydraulic configuration and feeding strategy strongly influenced the OCV dynamics of the VFCW–MFC systems (Figure 7). All configurations exhibited a characteristic transient OCV response, with a rapid increase following PWW feeding and a gradual decline over time. This behavior is typical of batch-fed VFCW–MFC and reflects the initial availability of biodegradable matter followed by substrate depletion and reduced electron donor availability. As OCV represents the potential difference between anode and cathode, its temporal profile provides insights into the establishment and maintenance of redox stratification within the system [32,44].

Fully saturated hydraulic conditions, as applied in VFCW–MFC 3′, are widely reported to restrict oxygen diffusion into the substrate, promoting the development of anoxic and anaerobic microenvironments along the vertical profile of CW systems [46,58,59]. Such redox conditions are considered favorable for the activity of electrogenic and denitrifying microbial communities. It should be noted, however, that dissolved oxygen and oxidation-reduction potential were not measured in the present study, and therefore, the establishment of redox stratification within the experimental columns cannot be directly confirmed.

Hydraulic operation markedly influenced the OCV response. VFCW–MFC 2′, comprising an unsaturated first stage (2.1′) followed by a saturated MFC stage (2.2′), produced moderate OCV peaks (~190–210 mV, Figure 7A) with a gradual decline, likely due to partial consumption of readily biodegradable organic matter during the first stage [32]. In contrast, the fully saturated VFCW–MFC 3′, produced the highest OCV values (~210–220 mV, Figure 7B), suggesting that continuous saturation may have promoted more stable redox conditions conducive to electron transfer toward the electrode, consistent with mechanisms reported in analogous CW–MFC systems [46,58]. Fractionated feeding in VFCW–MFC 4′ resulted in lower but more stable OCV values (~110–150 mV, Figure 7C), suggesting that dividing the daily hydraulic load attenuated transient voltage peaks and maintained a more uniform electrochemical response throughout the operational cycle [32,44,45]. Importantly, fractionated feeding did not increase peak OCV values but rather promoted a more stable electrochemical profile, indicating that its principal benefit was improved hydraulic and operational stability rather than enhanced peak electrochemical response. The 60–20 cm arrangement (deep anode, shallow cathode) consistently produced the highest OCV values, supporting the importance of maximizing vertical redox gradients that sustain high anoxia at the anode and facilitate cathodic oxygen access via surface diffusion and rhizosphere oxygenation [15,44].

Overall, the results demonstrate that hydraulic configuration, including saturation, feeding regimes, and electrode placement, strongly influence the OCV response of VFCW–MFC systems by regulating substrate availability and redox stratification. The highest peak OCV was associated with saturated operation, whereas fractionated feeding promoted a more stable electrochemical profile rather than higher peak voltages. As already stated, OCV does not directly quantify recoverable electrical power, but can be a useful indicator of the electrochemical conditions established within the CW.

### 3.3. Microalgae Growth and Effluent Treatment

**Figure 8:**
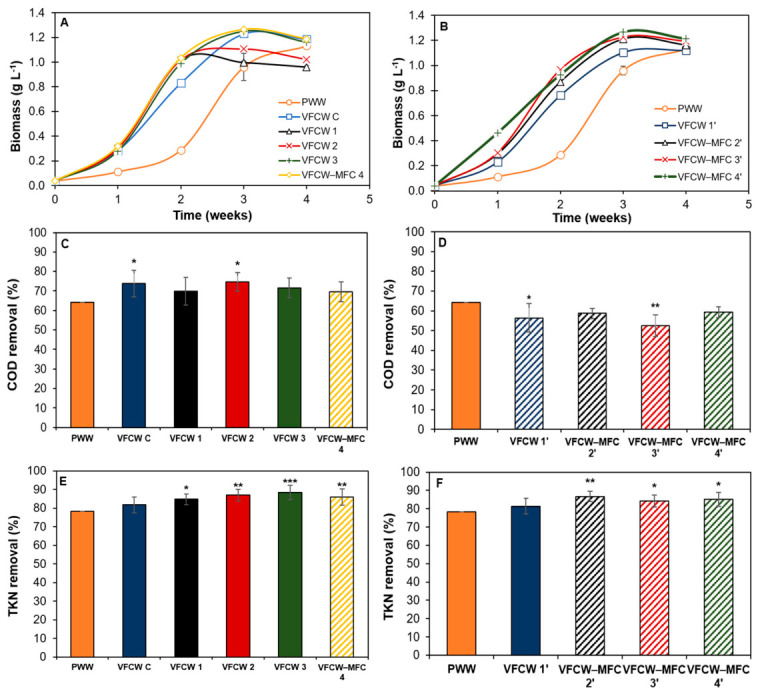
Microalgal biomass production and pollutant removal using effluents from vertical flow constructed wetlands (VFCWs) coupled with microbial fuel cells (MFCs): Algal growth curve in the first (**A**) and second (**B**) assays; Chemical Oxygen Demand (COD) removal in the first (**C**) and second (**D**) assays; Total Kjeldahl Nitrogen (TKN) removal in the first (**E**) and second (**F**) assays. VFCW configurations (first assay): VFCW C (C.1–C.2)—unplanted with passive aeration (Control); VFCW 1 (1.1–1.2)—both stages planted and non-aerated; VFCW 2 (2.1–2.2)—planted and aerated first stage (2.1) followed by a planted, non-aerated second stage (2.2); VFCW 3 (3.1–3.2)—both stages planted and aerated; VFCW–MFC 4 (4.1–4.2)—both stages planted, aerated, and integrated with microbial fuel cells (MFCs) with only unit 4.2 operated under saturated conditions. VFCW configurations (second assay): VFCW 1′ (1.1′–1.2′)—first stage operated under unsaturated conditions (1.1′), followed by a saturated second stage without MFC (1.2′); VFCW–MFC 2′ (2.1′–2.2′)—first stage unsaturated (2.1′), followed by a saturated second stage equipped with an MFC (2.2′); VFCW–MFC 3′ (3.1′–3.2′)—both stages operated under saturated conditions, with an MFC installed in the second stage (3.2′), fed with a single pulse of 2 L. VFCW–MFC 4′ (4.1′–4.2′)—same configuration as VFCW 3, but fed in a fractionated mode (4 × 500 mL). All systems of the second assay were planted and operated under passive aeration. Values are given as the average over the 4-week assay. Asterisks indicate significant differences between PWW according to Dunnett’s test (* *p* < 0.05; ** *p* < 0.01; *** *p* < 0.001).

Microalgal cultivation assays using VFCW-treated effluents exhibited a consistent pattern: biomass accumulation until approximately week 3, followed by stabilization by week 4 (Figure 8A,B).

In the first assay (Figure 8A), raw piggery wastewater exhibited an initial two-week lag phase, achieving 0.287 g L^−1^ through week 2, whereas post-VFCW effluents achieved 0.99–1.03 g L^−1^ by the same week, highlighting a kinetic advantage for pretreated substrates [27,60]. By week 3, VFCW 3 and VFCW–MFC 4 attained 1.250 and 1.264 g L^−1^, respectively, *versus* 0.960 and 0.997 g L^−1^ for PWW and VFCW 1; week 4 showed plateaus or modest declines across treatments. In the second assay (Figure 8B), a similar pattern was obtained, with VFCW–MFC 4′ having the highest biomass concentration in week 1 (0.462 g L^−1^), among other conditions, and reaching maximal concentration (1.267 g L^−1^) by week 3, compared to PWW (0.960 g L^−1^). Although pronounced differences were found in the initial growth stages, by the end of the cultivation period, biomass concentrations were within the range of 1.1–1.3 g L^−1^ across treatments.

These findings evidence that VFCWs can effectively act as “media conditioners” for *Tetradesmus obliquus* cultivation by reducing turbidity, coloration, and inhibitory substances that hinder the establishment of microalgae cultures [60,61]. The main benefit of the pretreatment was an accelerated growth phase and improved culture stability rather than a substantial increase in final biomass, which reached similar levels among treatments. Raw wastewater (PWW), despite achieving 1.132 g L^−1^, ultimately displayed a two-week lag phase attributable to optical constraints and algal-bacterial instability in its organic matrix, rather than nutrient scarcity, a dynamic prevalent in untreated wastewaters [62].

The weeks 3–4 plateau reflects self-shading at ~1.0 g L^−1^, reducing irradiance (52 µmol m^−2^ s^−1^), alongside pH elevation driving NH_4_^+^/NH_3_ shifts for assimilation or volatilization under aeration (1 vvm) [56,61]. This sequence of rapid initial growth yielding to light/nutrient limits exemplifies phycoremediation dynamics, with microalgae assimilating N/P and organics into biomass while bacteria aid COD transformation [63].

Regarding remediation performance, COD removal during microalgal cultivation depended on the characteristics of the VFCW-treated effluent rather than showing a uniform improvement over raw PWW. In the first assay (Figure 8C), COD removal efficiencies ranged from 64.2% in raw PWW to 69.7–74.7% across the VFCW effluents, indicating that the pretreatment improved medium quality and favored subsequent organic matter conversion during microalgae growth. Dunnett’s test revealed that VFCW C (73.7%; *p* = 0.037) and VFCW 2 (74.7%; *p* = 0.019) achieved significantly higher COD removal than PWW.

In the second assay (Figure 8D), COD removal efficiencies in the VFCW–MFC systems were comparatively lower, varying between 52.5% and 59.4%. Dunnett’s test indicated that VFCW–MFC 3′ (52.5%; *p* = 0.002) and VFCW 1′ (56.4%; *p* = 0.037) exhibited significantly lower COD removal than PWW. This behavior suggests that more extensive upstream VFCW–MFC pretreatment removed a substantial fraction of the readily biodegradable organic matter, thereby reducing the bioavailable carbon for mixotrophic assimilation by the microalgae. VFCW–MFC 4′ exhibited the highest removal among the coupled systems (59.4%) but did not differ significantly from PWW. Therefore, the comparatively lower COD removals during the microalgal stage should not be interpreted as reduced overall treatment performance but rather a consequence of the upstream process modifying the quantity and composition of the residual organic matter available for algal metabolism [64].

In contrast, TKN removal remained consistently high across all treatments (Figure 8E–F), demonstrating the robustness of nitrogen remediation during *T. obliquus* cultivation. In the first assay, TKN removal increased from 78.1% in raw PWW to 81.7–88.4% in VFCW-treated effluents. Dunnett’s test confirmed that four out of five pretreated effluents achieved significantly higher TKN removal than PWW: VFCW 1 (84.8%; *p* = 0.022), VFCW 2 (86.9%; *p* = 0.002), VFCW 3 (88.4%; *p* < 0.001), and VFCW–MFC 4 (85.9%; *p* = 0.006). Only the unplanted control VFCW C (81.7%; *p* = 0.355) did not differ from PWW, underscoring the role of plant presence in conditioning nitrogen for downstream phycoremediation. Similarly, in the second assay, all VFCW–MFC systems achieved elevated TKN removals (81.3–86.7%), exceeding the performance of raw PWW (78.1%). Dunnett’s test revealed that three VFCW–MFC effluents yielded significantly higher TKN removal than PWW: VFCW–MFC 2′ (86.7%; *p* = 0.005), VFCW–MFC 3′ (84.2%; *p* = 0.050), and VFCW–MFC 4′ (85.1%; *p* = 0.021). Notably, VFCW 1′ (81.3%; *p* = 0.461) did not differ significantly from PWW. These results reinforce the role of integrated VFCW–MFC pretreatment in conditioning nitrogen species for subsequent microalgal assimilation. These removals likely resulted from the combined effects of nitrogen assimilation into algal biomass, bacterial nitrification–denitrification, and ammonia volatilization promoted by photosynthetically induced pH elevation under continuous aeration [65,66].

### 3.4. Treatment Performance of the Integrated VFCW–MFC–PBR

**Figure 9:**
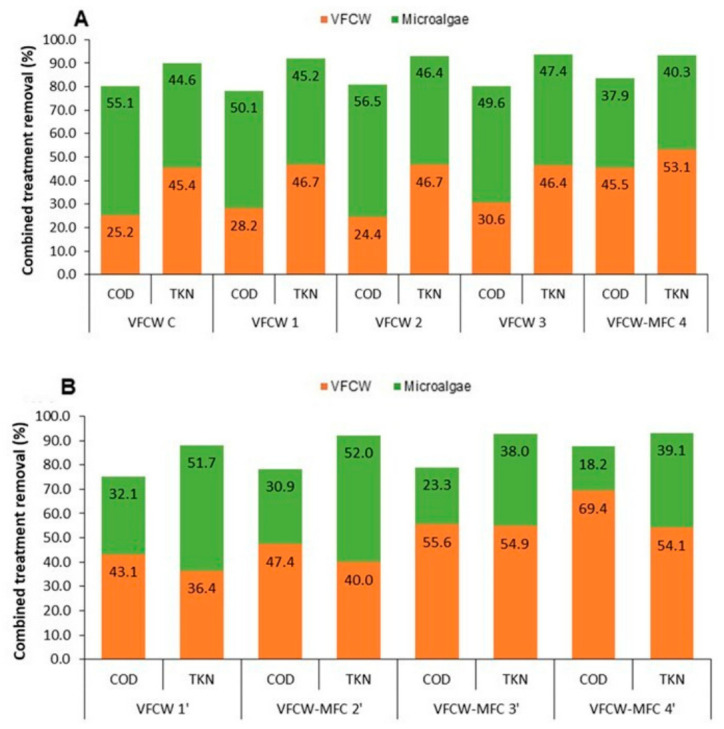
Combined Chemical Oxygen Demand (COD) and Total Kjeldahl Nitrogen (TKN) removal efficiencies in assays integrating Vertical Flow Constructed Wetlands (VFCWs) with microalgae growth. (**A**) First assay. VFCW configurations: VFCW C (C.1–C.2)—unplanted with passive aeration (Control); VFCW 1 (1.1–1.2)—both stages planted and non-aerated; VFCW 2 (2.1–2.2)—planted and aerated first stage (2.1) followed by a planted, non-aerated second stage (2.2); VFCW 3 (3.1–3.2)—both stages planted and aerated; VFCW–MFC 4 (4.1–4.2)—both stages planted, aerated, and integrated with microbial fuel cells (MFCs) with only unit 4.2 operated under saturated conditions. (**B**) Second assay. VFCW configurations: VFCW 1′ (1.1′–1.2′)—first stage operated under unsaturated conditions (1.1′), followed by a saturated second stage without MFC (1.2′); VFCW–MFC 2′ (2.1′–2.2′)—first stage unsaturated (2.1′), followed by a saturated second stage equipped with an MFC (2.2′); VFCW–MFC 3′ (3.1′–3.2′)—both stages operated under saturated conditions, with an MFC installed in the second stage (3.2′), fed with a single pulse of 2 L. VFCW–MFC 4′ (4.1′–4.2′)—same configuration as VFCW 3, but fed in a fractionated mode (4 × 500 mL). All systems of the second assay were planted and operated under passive aeration.

The integration of VFCW with a subsequent microalgal cultivation stage establishes a robust synergistic system for wastewater remediation. Regarding the cumulative COD and TKN profiles, VFCW effluent outputs in the second assay delivered lower residual COD (Figure 9B) than in the first one (Figure 9A), while TKN remained elevated across both assays (Figure 9). This pattern aligns with the observation that in the second assay, there was a more rapid initiation in specific treatments, particularly in VFCW–MFC 4′. Such results suggest that cumulative COD reduction along the treatment chain (VFCW → microalgae) primarily improved medium transmittance and reduced initial heterotrophic competition [67]. High concentrations of readily bioavailable organic carbon (COD > 1000 mg L^−1^) often promote heterotrophic bacterial dominance over photosynthetic microorganisms, leading to unstable algal-bacterial dynamics [67,68]. Conversely, the eventual convergence of final biomass between treatments and assays implies a physiological ceiling imposed by limited internal irradiance and the depletion of easily assimilable nitrogen sources. This cumulative behavior indicates that performance gains depend less on additional nutrient availability and more on the reduction in optically active organic fractions, where COD is correlated with color and turbidity, and the stabilization of inorganic conditions to improve microalgae growth [62,69].

A critical factor in this kinetic variation is the heterogeneity of nitrogen speciation at the VFCW outlets, where high ammonia often coexists with elevated nitrate. Incomplete nitrification in the VFCW stage directly contributes to the nutrient profile of the algal stage [27,35]. Microalgae preferentially assimilate ammonium due to its lower energetic cost, as it can be directly incorporated into amino acids, while nitrate must first be reduced to ammonium through enzymatic reactions requiring reducing power [61]. However, under uncontrolled photosynthetic activity, the resulting elevation in pH, combined with active aeration, promotes substantial ammonia nitrogen loss via volatilization [56,61]. This forced transition to nitrate assimilation can influence the specific growth rate and explains why certain treatments demonstrated superior early biomass (immediate availability coupled with improved optical conditions) yet converged by the end of the cycle due to self-shading and accelerated ammonia loss [62,67]. In these phycoremediation systems, nitrogen removal is intrinsically coupled with pH dynamics, nitrogen speciation, and competitive interactions within the algal-bacterial consortia [61,63].

These findings corroborate the efficacy of the VFCW followed by microalgae cultivation as an integrated treatment strategy for piggery wastewater. The VFCW works as a primary “medium conditioner”, reducing turbidity, ammonia, and potential inhibitory compounds, thereby creating favorable conditions for subsequent microalgal growth. The microalgal stage then provides an effective tertiary polishing step, particularly for nitrogen removal, producing a valuable active biological biomass [61,69]. Microalgae possess superior carbon fixation and nutrient assimilation capacities compared to higher plants, enabling the removal of refractory organic compounds that microbial communities in CWs struggle to degrade [62,70]. Observations further identify two primary optimization pathways for large-scale application: (i) pH control, potentially minimizing ammonia volatilization and sustaining photosynthesis [62,71]; and (ii) the further reduction in optically active COD fractions through polished filtration or refined CW operation to accelerate culture initiation and maximize light penetration [33,67].

### 3.5. Limitations and Future Research

The present study provides a proof-of-concept evaluation of an integrated Vertical Flow Constructed Wetland–Microbial Fuel Cell–Photobioreactor (VFCW–MFC–PBR) system for piggery wastewater treatment and resource recovery. The results demonstrate the feasibility of combining these technologies to improve pollutant removal, promote bio-electrochemical activity, and enhance microalgal biomass production. Nevertheless, these findings should be interpreted within the scope of the proof-of-concept and sequential optimization strategy adopted in this work, and several limitations should be acknowledged.

The VFCW–MFC configurations were evaluated using single custom-built two-stage reactors, with repeated measurements collected after stable operating conditions had been established. Consequently, the observed differences among configurations should be interpreted as comparative operational responses within the sequential optimization strategy adopted in this work. Future studies incorporating independently replicated reactor systems would further strengthen and validate these observations.

The bio-electrochemical performance of the integrated system was assessed through OCV measurements, which provided comparative indicators of redox stratification and electrogenic activity but did not directly quantify recoverable electrical power. Likewise, the establishment of redox gradients and the proposed pollutant removal pathways were inferred from system performance and the established behavior of analogous CW–MFC systems rather than from direct electrochemical, dissolved oxygen, oxidation–reduction potential, or microbiological characterization. Future studies incorporating comprehensive electrochemical analyses, vertical redox profiling, and microbial community characterization will contribute to a more detailed mechanistic understanding of the processes governing integrated VFCW–MFC operation and nitrogen transformation pathways.

The hydraulic behavior of the saturated wetland layers was inferred from the operational configuration adopted in this study, while the physicochemical characterization of the packing material was limited to particle size distribution. Future investigations can incorporate hydraulic tracer studies and more detailed substrate characterization to further elucidate the influence of flow patterns and media properties on treatment performance and phosphorus retention. In addition, aluminum was selected as the anodic material based on practical considerations, and the potential influence of anodic corrosion and dissolved aluminum release on phosphorus dynamics was not specifically evaluated and needs further investigation.

Although the integrated VFCW–MFC–PBR system demonstrated effective pollutant removal and improved microalgal cultivation, longer-term operation is required to evaluate substrate aging, phosphorus retention capacity, electrode durability, and seasonal variability under practical operating conditions. Further research should also investigate the optimization of hydraulic loading and operational strategies, as well as the agronomic and environmental performance of the recovered biomass and treated effluent.

Importantly, the scalability and practical applicability of this integrated circular bioeconomy approach are currently being assessed through pilot- and field-scale validation in India within the framework of the bilateral Portugal–India WCAlgaeKIT+ project (DRI/India/0609/2020), providing an important step towards the implementation of decentralized and sustainable livestock wastewater management systems.

## 4. Conclusions

The integrated VFCW–MFC–PBR system proved effective for simultaneous wastewater treatment, bio-electrochemical activity, and microalgal biomass production. MFC integration provided additional functionality via measurable bio-electrochemical responses. Hydraulic saturation enhanced the OCV response, reaching a maximum of 539 mV, while fractionated feeding promoted greater operational stability and performance.

VFCW conditioning was essential for improving the suitability of piggery wastewater for *Tetradesmus obliquus* cultivation by reducing turbidity, coloration, and inhibitory compounds, thereby enhancing early growth kinetics. By reducing the lag phase adaptation of *T. obliquus*, the treatment achieved higher removal rates during the initial growth stages and slightly higher biomass concentration compared to raw piggery wastewater.

The proposed system also demonstrated effective phycoremediation performance, achieving substantial COD and TKN removals alongside biomass generation. Nitrogen removal remained consistently high across treatments, while COD removal was more dependent on the composition and bioavailability of residual organic matter.

Importantly, integrating VFCW–MFC–PBR systems as circular bioeconomy platforms that couple wastewater remediation, bio-electrochemical activity, and biomass valorization may represent a sustainable and low-cost solution for rural communities in countries such as India, where infrastructure for managing livestock wastewater is often limited. Their low energy demand, operational simplicity, and decentralized nature make these systems particularly attractive for improving sanitation, reducing environmental pollution, and supporting sustainable agricultural practices while recovering valuable resources from waste streams.

## Figures and Tables

**Table 1:** Composition of the two effluent batches used in the study: total suspended solids (TSS), total volatile solids (TVS), chemical oxygen demand (COD), total Kjeldahl nitrogen (TKN), total ammoniacal nitrogen (TAN), nitrate (NO_3_^−^), and phosphate (PO_4_^3−^). Values are presented as mean ± standard deviation (*n* = 2).

Parameter	PWW 1	PWW 2
TSS (mg L^−1^)	1615 (±505)	1011 (±145)
TVS (mg L^−1^)	1135 (±15)	786 (±89)
COD (mg L^−1^)	3525 (±82)	4706 (±0)
TKN (mg L^−1^)	1092 (±0)	1232 (±28)
TAN (mg L^−1^)	903.0 (±7.0)	1183 (±7)
NO_3_^−^ (mg L^−1^)	140.0 (±22.1)	140.1 (±11.3)
PO_4_^3−^ (mg L^−1^)	41.50 (±1.77)	52.75 (±1.41)

## Data Availability

The original contributions presented in this study are included in the article. Further inquiries can be directed to the corresponding author.

## Abbreviations

The following abbreviations are used in this manuscript:

PWW	Piggery wastewater
VFCW	Vertical flow constructed wetland
MFC	Microbial fuel cell
PBR	Photobioreactor
TSS	Total suspended solids
TVS	Total volatile solids
COD	Chemical oxygen demand
TKN	Total Kjeldahl nitrogen
TAN	Total ammoniacal nitrogen
PVC	Polyvinyl chloride
OCV	Open circuit voltage
